# Development and psychometric evaluation of uncertainty about disease and treatment scale in hemodialysis patients: a sequential-exploratory mixed-method study

**DOI:** 10.1186/s40359-024-01685-x

**Published:** 2024-04-05

**Authors:** Sobhan Rahimi Esbo, Fatemeh Ghaffari, Zahra Fotokian, Hossein-Ali Nikbakht, Kiana Saadati

**Affiliations:** 1 Student Research Committee, Nursing Care Research Center, Health Research Institute, Babol University of Medical Sciences, Babol, I.R. Iran; 2 Nursing Care Research Center, Health Research Institute, Babol University of Medical Sciences, Babol, I.R. Iran; 3 Social Determinants of Health Research Center, Health Research Institute, Department of Biostatistics & Epidemiology, School of Public Health, Babol University of Medical Sciences, Babol, I.R. Iran; 4 Student Research Committee, Ramsar Campus, Mazandaran University of Medical Sciences, Ramsar, I.R Iran

**Keywords:** Uncertainty, Treatment, Disease, Hemodialysis, Scale, Psychometric evaluation

## Abstract

**Background and objective:**

The need for long-term treatment and frequent visits to treatment centers for hemodialysis can lead to psychological problems such as Uncertainty about Disease and Treatment (UC about D&T) in patients with chronic kidney failure. In order to understand uncertainty about disease and treatment and to plan for preventive measures and care interventions in various dimensions, there is a need for reliable and valid tools. The present study was conducted to design and psychometrically evaluate the Uncertainty about Disease and Treatment Scale (UC about D&TS) in patients undergoing hemodialysis.

**Methods:**

This study is of a methodological type and conducted in two stages. The first stage included a deductive (literature review) and an inductive approach (face-to-face interviews). In the second stage, psychometric indices of the UC about D&TS, including face validity (qualitative-quantitative), content validity (qualitative-quantitative), construct validity (exploratory factor analysis), and reliability (using Cronbach's alpha and McDonald's omega) were examined.

**Results:**

In the literature review stage, 66 items were extracted, and in the qualitative stage, 48 items were extracted. After merging similar items, 29 items were entered into the psychometric process. No items were removed in the face and content validity stages. In the construct validity stage, five factors were extracted, including self-uncertainty, uncertain situation, uncertain future, uncertainty of treatment outcomes, and information uncertainty, which constituted a total of 82.16% of the total variance. In this stage, five items were removed from the study due to a corrected item-total correlation below 0.32, and four items were removed due to cross-loading. The **α** and **Ω** were calculated as 0.828 and 0.818, respectively. The measurement stability and standard error of measurement were estimated at 0.977 and 2.019, respectively.

**Conclusion:**

The results showed that the UC about D&TS is a valid and reliable measure for patients undergoing hemodialysis. This scale is specifically designed to measure UC about D&T in hemodialysis patients, and it is recommended that healthcare providers (Hcps) use this scale in follow-up visits.

## Introduction

Chronic kidney disease is a threatening condition for affected individuals [1]. According to the report of the Global Burden of Disease (GBD), this disease is predicted to be the fifth leading cause of death worldwide by 2040 [2]. Treatment methods for chronic kidney disease (CKD) include peritoneal dialysis, hemodialysis, and kidney transplant. In Iran, the number of patients with CKD was 320,000 in 2019, of whom 49% received kidney transplants, 48% underwent hemodialysis, and 3% underwent peritoneal dialysis [3].

Various studies in hemodialysis patients indicate that different factors significantly impact the quality of life and outcomes associated with hemodialysis. Hemodialysis, as a complex clinical condition, is also linked to the psychosocial well-being of these patients [4]. Fatigue, irritability, anxiety, depression, and feelings of sorrow and grief are among the psychological issues mentioned in various studies for these patients [5]. According to Guerra et al.'s study (2021), these psychosocial challenges may be correlated with the disruption of certain tests in these patients, such as creatinine levels [6]. Anxiety and concern in these patients may accompany a decline in functional status, and the level of self-perception in hemodialysis patients may be related to personality dimensions [7]. Moreover, according to the findings of the study conducted by Yonata et al. (2022), other factors such as economic status and comorbidities are also associated with the quality of life in these patients [8]. One of the psychological challenges in these patients is the uncertainty they experience. Although hemodialysis treatment increases survival and life expectancy, uncertainty about the disease and the need for ongoing hemodialysis cause various psychological consequences, such as Uncertainty about Disease and Treatment (UC about D&T) [9–11].

Uncertainty about disease is defined as the inability to determine the meaning of events related to the disease, in situations where the patient or their family is unable to evaluate events due to a lack of sufficient symptoms or to predict the consequences of the disease [12]. According to Sahaf et al. (2016), insufficient information, doubt, lack of change in the treatment process, and disease non-improvement can lead to uncertainty in hemodialysis patients. Fear of death puts hemodialysis patients in uncertain situations and prevents them from having a regular life plan. Lack of sufficient information, comorbidities, and high mortality rates put these individuals in uncertain conditions about their future [11]. Uncertainty is a significant source of stress in chronic disease [13]. Based on the results of Goyal et al. (2018), the greatest psychological challenge for patients undergoing hemodialysis treatment is uncertainty [14], which can decrease adherence to treatment regimens [15, 16]. Nevertheless, researchers and healthcare providers (Hcps) have predominantly overlooked this issue. To implement evidence-based interventions for cases of UC about D&T, Hcps need access to a specific, valid, and reliable tool. Thus far, only one tool has been designed to measure uncertainty about the disease, developed by Mishel (1981). This tool has 27 items in two dimensions and is used to measure uncertainty about chronic diseases [17]. However, this tool is generally designed for diseases and is not specifically for hemodialysis patients. Also, it only measures uncertainty about the disease and does not address treatment. Torres and Pena-Amaro (2015) translated this scale into Spanish and validated it for hemodialysis patients [18]. Therefore, even though this scale is specifically for hemodialysis patients, it only addresses the disease discussion and does not measure uncertainty about the treatment. In Iran, Sajadi (2014) translated and validated this tool for cancer patients [19]. Therefore, this tool is also not specifically for hemodialysis patients. Designing a tool to measure UC about D&T specifically for patients undergoing hemodialysis is a necessity, which has not been addressed to date. The present study was designed to develop such a tool, and to evaluate its psychometric properties.

## Materials and methods

This study is a methodological research that was conducted in 2022-2023. The study was carried out in two stages, including: 1) a qualitative stage to generate items; and 2) a quantitative stage to examine the psychometric properties of the scale.

### Item generation

This stage was conducted in two steps. The first step involved reviewing the literature, and the second step included interviewing hemodialysis patients.

### Literature review

Electronic English databases including PubMed, Scopus, ISI Web of Science, as well as Persian databases including MagIran, SID, and Iran Medex were searched using the keywords “uncertainty,” “treatment,” “disease,” “hemodialysis,” “dilemma,” “scale,” “questionnaire” with no time limit. The selection criteria for the study included full access to articles in English or Persian. In this stage, nine English and three Persian articles (ten quantitative and two qualitative studies) were obtained. Text analysis was conducted, and codes related to uncertainty were extracted. A pool of 83 items was generated, and the items were reviewed by the research team. Similar items were combined into one.

### Interview with participants

In this stage, face-to-face and semi-structured interviews were conducted with 12 hemodialysis patients. The samples were purposefully selected, and efforts were made to ensure maximum diversity in personal and social characteristics of the interviewees, such as age, education level, gender, marital status, and place of residence. Sampling continued until data saturation was reached, i.e., when the researcher did not encounter any new cases in data analysis due to repetition of codes and the lack of formation of new subcategories and categories. Interviews were conducted in a quiet room adjacent to the hemodialysis unit and were scheduled upon participant request after completing a hemodialysis session. Participants who were invited for the interview had the ability to express their experiences and perspectives regarding their uncertainty about disease and treatment. The average duration of interviews was between 30 to 40 minutes. The interviews were conducted by one of the research team members (the corresponding author). An example of the interview questions is as follows: How would you describe your disease and treatment? What threatens the future of your disease and treatment? What factors contribute to the stability or improvement of your disease and treatment? Can you talk about any discouraging or encouraging experiences related to your disease and treatment? During the interview, follow-up questions were asked based on initial responses of participants. After each interview, a qualitative content analysis method was used to analyze the data. The interviews were transcribed, and each interview was coded. All interviews were read multiple times by the research team, and relevant codes were extracted from the text. In the next stage, inappropriate and repetitive codes were removed, and the remaining codes were visually edited. After repeated reading of the codes and identifying their similarities and differences, similar codes were grouped into one category. As the process of analysis progressed, the relationships between the categories became apparent, and the extracted categories were organized into themes. Based on the categories formed from the concept of uncertainty related to disease and treatment, the researchers extracted 48 items. An example of the item generation process is presented in Table 1.

**Table 1:** An example of the process of developing items in the uncertainty about disease and treatment in patients undergoing hemodialysis

**Participant experiences and opinions**	**Code**	**Item**	**Structure**
"Physicians have been saying from the very beginning that this disease is not curable, but manageable. The better you manage it, the longer you will live; but I see people who follow all the recommendations and still don't live long. I don't know if the things they tell us to eat or not eat will actually extend our lifespan or not?"	Uncertainty about living longer with adherence to treatment	I am not certain whether adhering to treatment will result in a longer lifespan	Uncertainty about treatment outcomes
"I doubt that I can find a job in the future. I don't know if my family will support me or not?"	Social damages caused by undergoing hemodialysis	I am not certain about having a favorable social or family status in the future due to my disease or hemodialysis.	Uncertain situation
"They told me once that my fistula was damaged, while I had followed all the care principles very carefully. I don't really know what to say. I thought to myself that maybe I don't have enough information about fistula care."	Uncertainty about information regarding vascular access care	I am not certain about my information regarding my vascular access.	Information uncertainty
"On the days that I undergo hemodialysis, I don't feel like doing any of my usual activities. The next one or two days, I feel a little better. I don't know if I will be able to do my activities in the future or not?"	Uncertainty about performing personal tasks due to hemodialysis	I am not certain about my ability to perform my daily activities considering the type of treatment I am receiving for my disease	Self-uncertainty
"Sometimes in the ward, I see patients who are feeling unwell and suffering a lot. I don't know what to do if I become involved in such a situation in a few weeks. Who should I ask for help?"	Uncertainty about the problems caused by treatment	Whenever an issue arises regarding my treatment, I find it difficult to make a decision about it easily.	Uncertain future

### Item reduction

At this stage, the psychometric properties of Uncertainty about Disease and Treatment Scale (UC about D&TS), including face and content validity (qualitative and quantitative), construct validity, and reliability were evaluated among patients undergoing hemodialysis. This scale was rated on a 5-point Likert scale, comprising strongly disagree, somewhat disagree, neither agree nor disagree, somewhat agree, and strongly agree.

#### Face validity

Face validity was evaluated using both qualitative and quantitative methods. In the qualitative method, the scale was sent to ten patients undergoing hemodialysis, asking them to provide feedback on the difficulty level, relevance, and ambiguity of items. Proposed modifications to the wording of the items were made based on their feedback. In the quantitative method, the scale was sent to ten hemodialysis patients (the same individuals who were invited to participate in the qualitative face validity evaluation) and they were asked to rate the importance of each item on a scale from 1 to 5 (1=not at all important, 2=somewhat important, 3=moderately important, 4=quite important, and 5=extremely important). The impact score of each item was then calculated using the following formula. The minimum impact score to retain an item was 1.5 [20].

$$\mathrm{Impact\ Score }=\mathrm{ Frequency }(\mathrm{\%})\times {\text{Importance}}$$

$$\mathrm{Frequency }\left(\mathrm{\%}\right):\mathrm{The\ number\ of\ individuals\ who\ gave\ a\ score\ of\ }4\ \mathrm{ or\ }5\ \mathrm{ to\ each\ item}$$

$${\text{Importance}}:\mathrm{The\ mean\ score\ of\ importance\ based\ on\ the\ Likert\ scale}$$

#### Content validity

Content validity of UC about D&TS was evaluated using both qualitative and quantitative methods. In the qualitative method, the scale was sent to ten nursing experts with experience in designing and validating tools, asking them to evaluate the scale in terms of grammar, wording, item allocation, and scaling. Based on their feedback, some of the items were revised. In the quantitative method, the content validity ratio (CVR) was calculated. In this stage, ten experts were asked to rate the necessity of each item on a 3-point Likert scale (1=not necessary, 2=useful but not essential, and 3=essential). The CVR was calculated using the formula [ne – (N/2)]/(N/2), "ne" being the number of experts who rated an item as 3 (essential), and N being the total number of experts. The results were compared to the Lawshe table, based on which the minimum acceptable value was determined to be 0.62 [21]. In this study, the CVR strict method was used, meaning only the essential items were included in the CVR formula.

After calculating the CVR, the content validity index (CVI) was calculated for the items. To do this, the same 10 experts who were invited to participate in the CVR evaluation were asked to rate each item on a 4-point Likert scale (completely relevant, relevant, somewhat relevant, and irrelevant). According to Lynn and colleagues (2007), the minimum acceptable level of CVI for a minimum of six experts is 0.78, which is considered excellent [22]. In this study, the minimum acceptable value for the CVI was considered to be 0.78. The S-CVI/UA and S-CVI/Ave indices were calculated based on the minimum acceptable value of 0.80 and 0.90, respectively [23, 24].

#### Item analysis

Before examining the construct validity, an item analysis was conducted to identify potential problems of the items by calculating the corrected item-total correlation. The correlation coefficient between the items less than 0.32 or greater than 0.9 was considered as the criterion for item deletion [25].

#### Construct validity

In this stage, a descriptive cross-sectional study was conducted. A total of 360 patients undergoing hemodialysis treatment in hemodialysis units of hospitals affiliated with Babol University of Medical Sciences, Iran, were invited to participate in the study using convenient sampling. Informed consent was obtained from the study participants. The inclusion criteria comprised being 18 years of age or older, history of a minimum of three months of hemodialysis treatment, no speech or hearing problems, and no history of kidney transplantation. Suffering from mental and cognitive disorders and reluctance to participate in the study were considered as exclusion criteria. To collect data from the sociodemographic and clinical questionnaire, variables such as age, gender, educational level, marital status, financial condition, occupation, insurance, place of residence, chronic disease, vascular access, duration of hemodialysis, number of months under hemodialysis treatment, and UC about D&TS were used.

The construct validity of the UC about D&TS was evaluated using exploratory factor analysis (EFA). The EFA was performed using the principal axis factor method with Promax rotation. The Kaiser-Meyer-Olkin (KMO) and Bartlett’s tests were performed to evaluate the adequacy and suitability of the sample. A KMO value greater than 0.7 was considered suitable [26]. In this study, factors were extracted based on Eigenvalues and the Scree Plot to determine the number of factors [27]. Factors with Eigenvalues above 1 were considered suitable and retained in the study. A factor loading of at least 0.3 was considered appropriate for assigning each item to a factor. This value was determined by the formula CV = 5.152 ÷ √ (n – 2), where CV is the minimum factor loading, and n is the sample size. Items with communalities less than 0.2 were excluded from the EFA [26].

#### Reliability

**Fig. 1:**
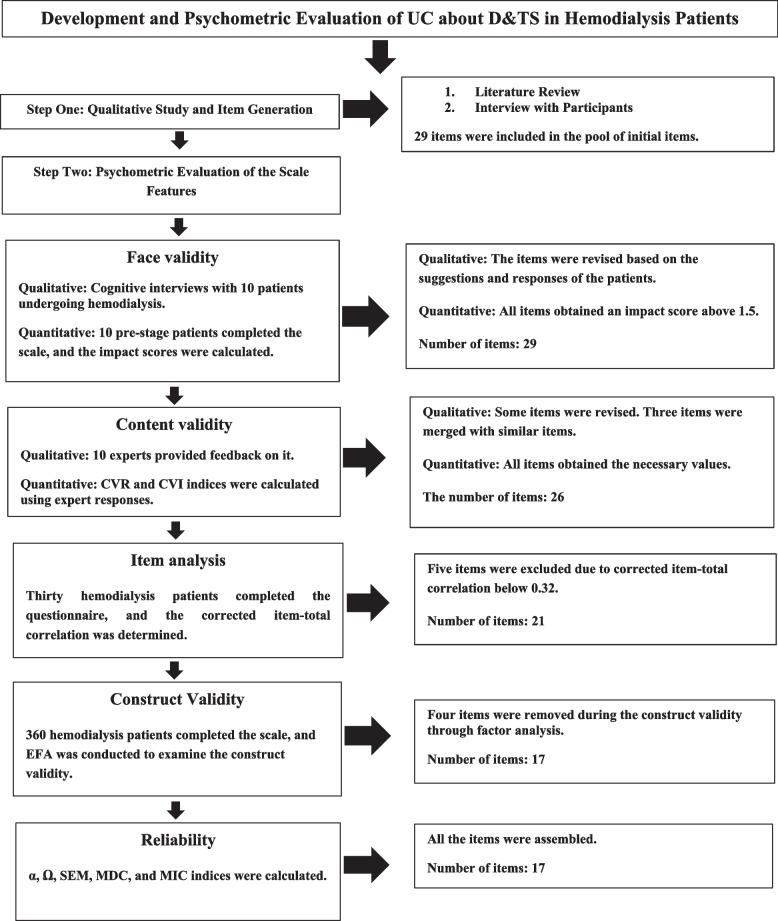
Development steps for UC about D&TS

The reliability of the scale was assessed using internal consistency and stability methods. Internal consistency was evaluated using Cronbach’s alpha (α), McDonald’s omega (Ω), and average inter-item correlation (AIC) indices. A value greater than 0.7 for α and Ω and an AIC above 0.2 were considered suitable for internal consistency [28]. In addition, absolute reliability was evaluated using the standard error of measurement (SEM) and the formula: SEM = SD Pooled × √ 1 − ICC [26]. Finally, the responsiveness of the scale was assessed using the Minimal Detectable Change (MDC) with the formula: MDC95% = SEM × √ 2 × 1.96 and the minimal important change (MIC) with the formula: MIC = 0.5 × SD of the Δ score. A MIC smaller than MDC indicates that the scale is responsive. The interpretability of the scale was also examined using the ceiling and floor effects and MDC [25]. Figure 1 depicts the steps involved in the design of the UC about D&TS.

### Ceiling and floor effects

The presence of ceiling and floor effects indicates that the items representing the maximum and minimum intensity of the phenomenon have not been included in the scale. The presence of ceiling and floor effects is demonstrated when 15% of the scores are at the maximum or minimum limit, meaning that 15% of responses provided by participants are at the minimum or maximum level of the phenomenon under study. This indicates the presence of a floor or ceiling effect [29].

### Scoring

The Likert scale was used to respond to the items in UC about D&TS. In the final version of the scale, a standardization method of 100 was used for scoring and comparing the scores of different dimensions of the scale. To convert the scores of the subscales and the total score to a range of 0 to 100, the following linear transformation formula was used [25]:

$$\mathbf{t}\mathbf{r}\mathbf{a}\mathbf{n}\mathbf{s}\mathbf{f}\mathbf{o}\mathbf{r}\mathbf{m}\mathbf{e}\mathbf{d} \mathbf\ {s}\mathbf{c}\mathbf{o}\mathbf{r}\mathbf{e}=\frac{\mathbf{a}\mathbf{c}\mathbf{t}\mathbf{u}\mathbf{a}\mathbf{l}\ \mathbf{r}\mathbf{a}\mathbf{w}\ \mathbf{s}\mathbf{c}\mathbf{o}\mathbf{r}\mathbf{e}-\ \mathbf{l}\mathbf{o}\mathbf{w}\mathbf{e}\mathbf{s}\mathbf{t} \ \mathbf{p}\mathbf{o}\mathbf{s}\mathbf{s}\mathbf{i}\mathbf{b}\mathbf{l}\mathbf{e}\ \mathbf{r}\mathbf{a}\mathbf{w} \ \mathbf{s}\mathbf{c}\mathbf{o}\mathbf{r}\mathbf{e}}{\mathbf{p}\mathbf{o}\mathbf{s}\mathbf{s}\mathbf{i}\mathbf{b}\mathbf{l}\mathbf{e}\ \mathbf{r}\mathbf{a}\mathbf{w} \ \mathbf{s}\mathbf{c}\mathbf{o}\mathbf{r}\mathbf{e}\ \mathbf{r}\mathbf{a}\mathbf{n}\mathbf{g}\mathbf{e}}\times 100$$

### Data analysis

The data was entered into SPSS 27 software. To examine the construct validity of UC about D&TS in hemodialysis patients, EFA with the PAF method was used. Additionally, to calculate the McDonald’s omega (Ω) and Cronbach’s alpha (α) coefficients, SPSS 27 software was used [30].

### Ethical considerations

The Ethics Committee of Babol University of Medical Sciences approved this research proposal (code IR.MUBABOL.HRI.REC.1400.229). All participants signed the written consent form and the rights of the participants were preserved, i.e., all data were kept anonymous and confidential.

## Results

The mean age of the 360 patients undergoing hemodialysis was 58.32 (±14.20). The mean duration of hemodialysis treatment was 40.21 (±50.05) months (Table 2).

**Table 2:** Sociodemographic and clinical characteristics of the study participants (*N*=360)

**Demographic characteristics**		**Frequency(percent)**
Gender	Female	155(43.1)
	Male	205(56.9)
Marital status	Single	27(7.5)
	Married	313(86.9)
	Divorced	5(1.4)
	Widowed	15(4.2)
Financial condition	Poor	93(25.8)
	Moderate	184(51.1)
	Good	83(23.1)
Occupation	Unemployed	99(27.5)
	Self-employed	84(23.3)
	Government employee	10(2.8)
	Retiree	51(14.2)
	Homemaker	112(31.1)
	Student	4(1.1)
Insurance	Yes	339(94.2)
	No	21(5.8)
Place of residence	Urban	159(44.2)
	Rural	194(53.9)
	Suburban	7(1.9)
Education level	Illiterate	112(31.1)
	Primary school	84(23.3)
	High school	48(13.3)
	Diploma	95(26.4)
	Bachelor	17(4.7)
	Master and doctorate	4(1.1)
Living situation	With spouse	148(41.1)
	With spouse and children	150(41.7)
	With children	24(6.7)
	With parents	22(6.1)
	With relatives	2(0.6)
	Alone	14(3.9)
Chronic disease	Yes	17(4.7)
	No	343(95.3)
Vascular access	Fistula	256(71.1)
	Shaldon	15(4.2)
	PermCath	84(23.3)
	Graft	5(1.4)
Hemodialysis duration	180 minutes	146(40.6)
	12- minutes	37(10.3)
	240 minutes	177(49.2)

### Item generation

The results of the literature review stage, which included 66 statements, and the results of the interviews, which included 48 statements, were reviewed multiple times by the research team. Inappropriate and repetitive statements were removed. Also, the final statements were edited several times in terms of appearance and grammar. At the end of the qualitative stage, 29 statements were preserved for entry into the psychometric stage.

### Item reduction

#### Face validity

In the qualitative method, some changes were made to the appearance of the statements based on recommendations of experts. The results of the quantitative face validity phase demonstrated all item scores to be greater than 1.5.

#### Content validity

Based on opinions provided by experts, three statements were merged in the qualitative content validity assessment, due to their similarity with other statements, resulting in 26 statements. In the quantitative content validity stage, all statements had CVR ≥ 0.62, CVI ≥ 0.78, and Kappa ≥ 0.75. Therefore, all statements were preserved. The values of S-CVI/UA and S-CVI/Ave were 0.84 and 0.98, respectively.

#### Construct validity

**Fig. 2:**
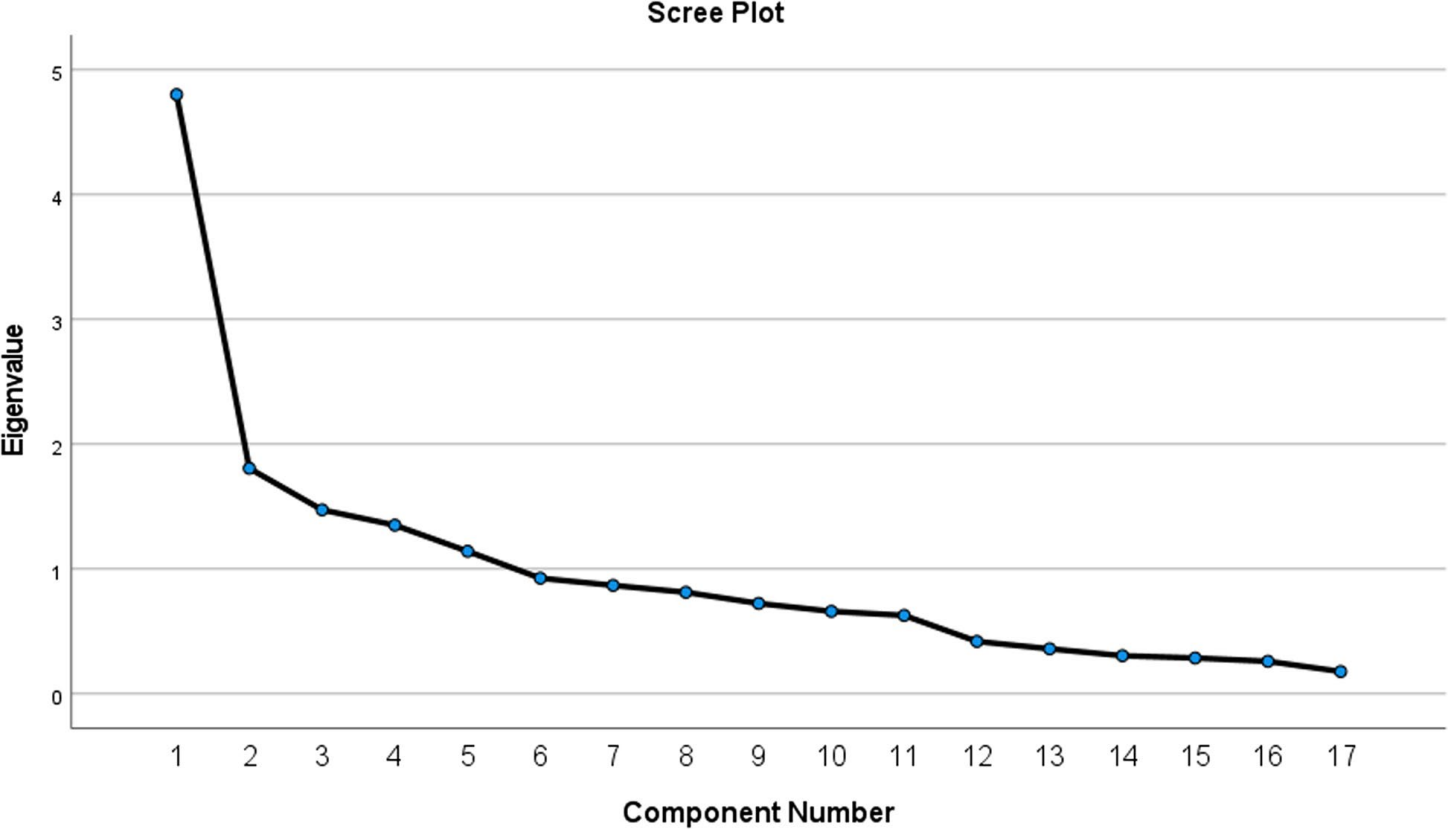
Scree plot for extracting factors in UC about D&TS

Before conducting the construct validity, a content analysis was performed. Five statements were removed due to the inter-item correlation being less than 0.32. In this stage, 21 statements were preserved for construct validity. To perform exploratory factor analysis, the Kaiser-Meyer-Olkin (KMO) test was conducted to ensure adequacy of the sample size. Subsequently, the Bartlett's test of sphericity was used to determine if the intercorrelations between variables were not equal to zero. The results showed that the KMO index was 0.715 and the Bartlett's test $({x}^{2}$=2133.960, p<0.01) was significant. In this model, five factors were extracted based on eigenvalues greater than 1 and scree plot (Fig. 2). Statements with factor loading ˂ 0.3, communalities ˂ 0.2, and cross-loading were excluded from the study. At the end of the construct validity stage, 17 statements remained in the scale. The five factors ultimately explained 82.16% of the variance. Table 3 shows the results of the construct validity and the variance of each factor.

**Table 3:** Results of exploratory factor analysis (EFA) for uncertainty about disease and treatment (*N*=360)

Factors	Item	Factor loading	h2*	M±SD	Skew (kurtosis)	λ**	%variance
Self-uncertainty	I am not certain about my ability to perform my daily activities considering the type of treatment I am receiving for my disease	0.875	0.440	10.46±4.6	0.15(-1.22)	3.799	22.34
	I am not certain if I can effectively utilize strategies to deal with the side effects associated with my treatment	0.829	0.578				
	I am not certain of my ability to be prepared to handle crisis situations related to hemodialysis	0.600	0.687				
	I am not certain if I can accept other treatment methods for my disease	0.390	0.310				
Uncertain situation	At times, when I become exhausted from the conditions associated with hemodialysis, I am unsure about continuing with it	0.880	0.596	10.86±4.6	0.16(-1.16)	3.087	18.15
	I am not certain that I can pursue my life goals and aspirations based on the type of treatment I am receiving.	0.559	0.633				
	I am not certain about having a favorable social or family status in the future due to my disease or hemodialysis	0.499	0.520				
	I am in a state where nothing in my life can be relied upon with certainty	0.473	0.361				
Uncertain future	I am not certain of what not to do in the future for my treatment	0.617	0.575	9.45±3.83	-0.01(-.94)	2.547	14.98
	Whenever an issue arises regarding my treatment, I find it difficult to make a decision about it easily	0.582	0.662				
	I have many unanswered questions about the future of my treatment	0.557	0.246				
Uncertainty of treatment outcomes	I am not certain whether hemodialysis is a suitable approach for extending my lifespan	0.989	0.733	6.97±3.49	0.71(-0.46)	2.511	14.77
	I am not certain whether educating the treatment team can reduce the complications of hemodialysis	0.384	0.301				
	I am not certain whether adhering to treatment will result in a longer lifespan	0.346	0.200				
Information uncertainty	I am not certain whether I have enough information about kidney transplantation or not	1.003	0.930	6.7±3.21	0.64(-0.5)	2.027	11.92
	I am not certain if a kidney transplant is better than hemodialysis	0.451	0.230				
	I am not certain about my knowledge of my vascular access information	0.437	0.279				

h2* =Communalities; λ** =Eigenvalue

#### Reliability

The stability of the scale was strong based on the ICC results. The absolute reliability was obtained as 2.019 according to the SEM result. This means that the scale score can vary by ± 2.019 in repeated measurements of an individual. Based on the MDC, MIC, ceiling and floor effects results, the scale is responsive. Moreover, the floor effect was 0.3% and the ceiling effect was 0.6%, indicating that the scale is exempt from these effects and is interpretable (Table 4).

**Table 4:** Results of the Reliability of the uncertainty about disease and treatment

Factors	ICC	CI95%	*P* Value	Ω	α	AIC	SEM	MDC	MIC
First	0.971	To 0.986 0.940	0.001>	0.742	0.727	0.399	0.784	2.175	0.835
Second	0.955	To 0.979 0.906	0.001>	0.708	0.703	0.373	0.977	2.708	0.968
Third	0.966	To 0.984 0.924	0.001>	0.734	0.678	0.407	0.623	1.72	0.653
Fourth	0.956	0.909 To 0.979	0.001>	0.601	0.588	0.322	0.732	2.029	0.746
Fifth	0.930	0.853 To 0.966	0.001>	0.722	0.623	0.352	0.851	2.358	0.882
Total	0.977	0.951 To 0.989	0.001>	0.818	0.828	0.219	2.019	5.596	1.998

### Scoring

The final version of the UC about D&TS consists of 17 items and 5 factors. The items were rated on a 5-point Likert scale (strongly agree=5, somewhat agree=4, neither agree nor disagree =3, somewhat disagree=2, strongly disagree=1), with the total score ranging between 17-85. None of the items were reverse-scored. Finally, the scale score is expressed from 0 to 100 using a linear transformation formula. A lower score indicates less uncertainty, while a higher score suggests more uncertainty.

## Discussion

In this study, UC about D&TS was designed in a deductive and inductive way, and its face, content and construct (exploratory factor analysis) validities; internal consistency; and stability were investigated. The findings of this study are comparable to those of the study by Mishel. However, Mishel's (1981) tool is only applicable for measuring uncertainty about disease, while UC about D&TS measures uncertainty about both disease and treatment.

The survival of hemodialysis patients is dependent on their adherence to treatment regimen and self-care [31, 32]. Specifically, uncertainty about treatment, may lead to negative consequences such as despair, confusion, and increased dialysis-related complications [13, 16]. Therefore, recognition of patient uncertainty about treatment by Hcps can lead to timely and effective evidence-based interventions.

The present study scale is also comparable to a study by Torres (2015). This scale was translated and psychometrically tested for hemodialysis patients; however, the item level S-CVI/Ave index obtained was 0.7, which is lower compared to the S-CVI/Ave in the present study (0.98) [18]. This may be due to the specific items of the present study being more appropriate for hemodialysis patients in terms of content.

Furthermore, content validity indexes were not reported quantitatively in Mishel's study [17]. To investigate the construct validity, factor analysis with Varimax rotation was used in Mishel's study, resulting in two factors that explained 35.9% of the total variance. Similarly, the study by Torres (2015) also used principal component analysis with Varimax rotation to investigate the construct validity, resulting in two dimensions (ambiguity and complexity), and explaining 36% of the total variance. In the present study, the construct validity was assessed using exploratory factor analysis with Promax rotation, resulting in 17 items and five factors that explained 82.16% of the total variance, which is much higher than the previous studies. UC about D&TS addresses dimensions of uncertainty in these patients that previous studies did not cover, explaining more variance in patient responses. In Mishel's study, the alpha coefficient was 91% for the first factor and 64% for the second factor, and other reliability indices were not reported [17]. In the study by Torres, the alpha coefficient for the entire scale was 0.72 [18]. However, in the present study, the alpha coefficient was 0.828. Comparison with Torres's study confirms the internal consistency of the present scale. In addition, unlike the previous two studies, the ICC was 0.977 and the Ω was 0.818, indicating good reliability.

Based on the results of this study, UC about D&TS included five factors of self-uncertainty, uncertain situation, uncertain future, uncertainty about treatment outcomes, and information uncertainty. The first factor (self-uncertainty) comprised four items that explain 22.34% of the total variance. This factor measures patient uncertainty about the disease and treatment. In fact, self-uncertainty deals with the individual aspects of disease and treatment, which have not been addressed in other studies [17, 18]. However, in the study conducted by Santana et al. (2020), the dimensions of self-care in hemodialysis patients were identified. One of the categories identified in this study was shortcomings in self-care. In this section, patients referred to two subthemes: "Transgressions in Self-care" and "Vulnerability to perform Self-care," which led to the formation of this category [33]. In the current study, the first factor also bears relative similarity to this category, and patients in the present study also acknowledged their challenges in this regard. This factor explains the highest amount of variance in the scale. The second factor (uncertain situation) included four items that explain 18.15% of the total scale variance. This factor deals with the social conditions and aspects of the individual and measures patient uncertainty about the disease and treatment in relation to these conditions. Considering that UC about D&TS has comprehensively addressed both treatment and disease conditions, this scale is preferred over other similar scales. The third factor (uncertain future) measures uncertainty of patients about future problems. Patients undergoing hemodialysis often experience uncertainty about their future due to the complexity of the treatment process, its psychological and social impacts, as well as financial constraints associated with the hemodialysis treatment [34]. This dimension conceptually has some relative similarity to the “complexity” factor in Torres’s study [18]. In addition, this factor is similar to the first dimension of the Sahaf study. In this study, the first dimension is 'Obscure Future,' which deals with uncertainty regarding the future of hemodialysis patients [11]. The uncertain future factor has three items and explains 14.98% of the total variance. The fourth factor (uncertainty of treatment outcomes) is a new concept that has not been addressed in other studies [17, 18]. Comprising three items, this factor explains 14.77% of the total tool variance, and measures uncertainty of patients about the results of treatment (hemodialysis) and their education. The reason for this difference is that the aspect of treatment has not been studied in the Mishel scale. The uncertainty of treatment outcomes in hemodialysis patients refers to the doubts and concerns patients have regarding the prediction and effects of hemodialysis treatment. This uncertainty can impact patients' decision-making, behaviors, and mental well-being, highlighting the need for improving communication between physicians and patients and providing better information for optimal treatment management [35]. The last factor (information uncertainty) embraces uncertainty of patients regarding their knowledge and information about the disease and treatment, which conceptually has some relative similarity to the "ambiguity" factor in the studies by Michel and Torres [17, 18, 36]. In the Sahaf study, the concept of insufficient information about hemodialysis treatment was addressed within the "obscure future" dimension [11]. However, in this study, despite a more comprehensive exploration of the uncertainty experienced by these patients, this concept has been considered with three items as contributing factors. This factor has three items that explain 11.92% of the total scale variance. In this dimension, the item "I am not certain whether I have enough information about kidney transplantation or not?” had the highest amount of factor loadings among all the scale items, indicating the importance of this issue in hemodialysis patients.

## Conclusion

The results of this study showed that UC about D&TS in patients undergoing hemodialysis comprises five factors. Encompassing 17 items, this scale has appropriate validity and reliability. Therefore, due to the low number of items and simplicity, it is recommended for use in investigating UC about D&T in patients undergoing hemodialysis by HCPs in follow-up visits.

## Research strengths

The present study is the first to design and psychometrically assess a specific tool for measuring UC about D&T in hemodialysis patients. Using a standard approach, including item generation for designing the items, as well as measuring the psychometric indices of the tool, is another strength of this study.

## Research limitations

This study was conducted in one of the northern cities of Iran. It is possible that access to healthcare services and the level of HCP education provided to patients, as well as other factors affecting UC about D&T, differ from other parts of Iran. Another limitation of this study is that the participants were invited to cooperate through convenience sampling; which may limit the generalizability of results.

## Data Availability

No datasets were generated or analysed during the current study.
